# Mitochondrial Calcium Dysregulation and Targeted Therapies in Heart Failure

**DOI:** 10.31083/RCM46211

**Published:** 2026-03-17

**Authors:** Mengting Liu, Yunpeng Jin

**Affiliations:** ^1^Department of Cardiology, The Fourth Affiliated Hospital of School of Medicine, and International School of Medicine, International Institutes of Medicine, Zhejiang University, 322000 Yiwu, Zhejiang, China

**Keywords:** heart failure, mitochondria, calcium, targeted therapy, mitochondrial calcium uniporter

## Abstract

Heart failure (HF) is steadily increasing in prevalence and poses a major global health challenge, with substantial medical and economic burdens. HF represents the terminal stage of diverse cardiac disorders and is characterized by poor prognosis despite the availability of conventional pharmacological treatments, underscoring the urgent need for novel therapeutic approaches. Accumulating evidence highlights a strong association between HF and mitochondrial dysfunction, of which dysregulated mitochondrial calcium (mCa^2+^) homeostasis plays a pivotal role in disease pathogenesis. Ca^2+^ serves as an essential signaling messenger that regulates energy metabolism and also governs cell survival and myocardial contractility. Thus, this review introduces the mechanisms of mCa^2+^ uptake and efflux and the association of these processes with HF and emerging therapeutic strategies. We also discuss mCa^2+^ uniporter (MCU) inhibitors and Elamipretide, a mitochondria-targeted peptide. Collectively, this work provides novel insights and preclinical evidence supporting mitochondria-based interventions for HF.

## 1. Introduction

Heart failure (HF) is a major global health challenge, characterized by persistently high incidence and mortality rates [1]. The prevalence of HF continues to rise due to aging of the population, increases in cardiovascular risk factors, and improved survival through advances in medical care. It is estimated that over 64 million people worldwide are currently affected by HF, accounting for approximately 1–3% of the global population. HF significantly impairs the patients’ quality of life and imposes a substantial healthcare and economic burden on society due to its high mortality rate, frequent hospitalizations, and considerable treatment cost [1, 2, 3]. For patients with HF with reduced ejection fraction (HFrEF), the current standard of care is guideline-directed medical therapy (GDMT) [4]. Although the implementation of GDMT has been shown to reduce two-year mortality by up to 73%, its global utilization rate is less than 25% [1, 5]. The EMPEROR-Preserved trial demonstrated that treatment with the sodium-glucose co-transporter 2 inhibitor (SGLT2i) empagliflozin significantly reduced the composite risk of cardiovascular death or hospitalization in patients with HF with preserved ejection fraction (HFpEF). SGLT2i is therefore an established first-line option for HFpEF [6, 7].

Mounting evidence suggests a close association between HF and mitochondrial dysfunction, with the clinical manifestations reflecting impaired energy metabolism [8]. Mitochondria are the primary generation site for adenosine triphosphate (ATP), the cellular energy source [9]. Moreover, mitochondria regulate cytosolic calcium (cCa^2+^) homeostasis, oxidative stress, and apoptosis, thereby playing a central role in maintaining metabolic balance and cell survival [10, 11].

Ca^2+^ is a critical second messenger in eukaryotic cells, participating in diverse physiological processes such as muscle contraction, neuronal excitation, and protein regulation [12, 13]. Mitochondria can dynamically regulate cCa^2+^ homeostasis through Ca^2+^ uptake and release [14]. The mitochondrial Ca^2+^ concentration (m[Ca^2+^]) directly influences ATP synthesis, opening of the mitochondrial permeability transition pore (mPTP), and broader Ca^2+^ signaling pathways [15]. Optimal m[Ca^2+^] promotes efficient ATP generation, while excessive Ca^2+^ induces mitochondrial dysfunction and impaired energy metabolism [16]. Dysregulated mitochondrial Ca^2+^ (mCa^2+^) homeostasis has been associated with the pathogenesis and progression of various diseases, including HF [17, 18]. Given the crucial role of mCa^2+^ in cellular physiological processes, it is essential that cells maintain mCa^2+^ homeostasis. Targeting the molecular mechanisms of mCa^2+^ regulation is therefore considered to be a highly promising strategy for the treatment of HF. This review summarizes the regulatory mechanisms of mCa^2+^ and their association with the development of HF. Furthermore, we introduce several recent advances in mitochondria-targeted therapeutic approaches for HF.

## 2. Regulation of Mitochondrial Calcium

The mitochondrial uptake and release of Ca^2+^ in cardiomyocytes is a fundamental biological process, with Ca^2+^ influx into mitochondria being essential for ATP production and contractile function. Mitochondria can adjust their handling of Ca^2+^ in response to cellular demands [19]. Physiologically, mitochondria do not retain Ca^2+^ indefinitely, but instead act as dynamic Ca^2+^ buffers. This prevents excessive fluctuation in the cytoplasmic Ca^2+^ concentration ([Ca^2+^]c), thereby safeguarding intracellular homeostasis [20, 21]. A key question raised by these observations is: what are the mechanisms by which Ca^2+^ is transferred across the mitochondrial membrane?

### 2.1 Mitochondrial Calcium Uptake System

Because mitochondria are double-membrane organelles, Ca^2+^ entry requires passage across both the outer mitochondrial membrane (OMM) and the inner mitochondrial membrane (IMM). The transfer of Ca^2+^ from the cytosol into the intermembrane space (IMS) is primarily mediated by voltage-dependent anion channels (VDACs), which serve as primary ion channels on the OMM [17].

#### 2.1.1 Voltage-Dependent Anion Channels

In mammals, VDACs exist in three isoforms: VDAC1, VDAC2, and VDAC3. Although VDAC1 is the most highly expressed VDAC in cardiac tissue, studies have shown that overexpression or knockout of any of these isoforms can affect mCa^2+^ uptake [22, 23, 24]. VDACs can also form macromolecular complexes with the Ca^2+^ channels of other organelles, thereby facilitating Ca^2+^ flux across the OMM. For instance, Szabadkai *et al*. [25] and Harada *et al*. [26] independently demonstrated that VDAC1 and VDAC2 can bind to inositol 1,4,5-trisphosphate receptor (IP3R) via the molecular chaperone glucose-regulated protein 75 (GRP75). Dysregulation of VDAC function has been implicated in various pathological conditions, including cardiovascular diseases [27].

#### 2.1.2 Mitochondrial Calcium Uniporter Complex

After entering the IMS, Ca^2+^ must traverse the IMM to reach the mitochondrial matrix. This process is mediated by the mitochondrial Ca^2+^ uniporter (MCU) complex [28]. Core components of the MCU complex in mammals include the MCU, the MCU dominant negative beta subunit (MCUb), the Essential MCU Regulatory Element (EMRE), and Mitochondrial Ca^2+^ Uptake protein 1/2 (MICU1/2) [29, 30]. These components are expressed in virtually all mammalian tissues [31]. Some of the core components of the MCU complex are briefly described in Table 1 (Ref. [29, 30, 32, 33, 34, 35, 36, 37, 38, 39]) below.

**Table 1.** **Core components of the MCU complex**.

Component name	Full name	Key characteristics & functions
MCU	Mitochondrial Ca^2+^ Uniporter	This pore-forming subunit is widely expressed in most mammals and is an essential component of the ion channel. Downregulation of MCU may affect Ca^2+^ uptake [29, 30, 32].
MICU1	Mitochondrial Ca^2+^ Uptake Protein 1	MICU1 is located in the IMS and is also widely expressed in most mammals, functioning as a Ca^2+^-sensing protein [33]. In the resting state, MICU1 plays a gatekeeper role by blocking access of Ca^2+^ to the MCU channel [34, 35].
MICU2	Mitochondrial Ca^2+^ Uptake Protein 2	MICU2 is distributed in visceral organs and is a paralog of MICU1 [34]. There is a general consensus that MICU1 and MICU2 modulate the process of Ca^2+^ permeation together, with the functional role of MICU2 being dependent on MICU1 [36, 37].
EMRE	Essential MCU Regulatory Element	EMRE is widely expressed in mammals and serves as an essential auxiliary subunit of the MCU. In the absence of EMRE, mitochondria may fail to efficiently uptake Ca^2+^, even when MCU is normally expressed [30]. This is supported by observations that Ca^2+^ permeation is significantly impaired in systemic knockout mouse models of EMRE, as well as in vitro cell experiments with specific EMRE knockdown [33, 36, 37].
MCUb	MCU dominant negative beta subunit	MCUb is a critical negative regulator whose primary function is to suppress Ca^2+^ uptake, thus preventing Ca^2+^ overload [38, 39]. It is abundantly expressed in cardiac and pulmonary tissues, and can interact with MCU [33].

Fan *et al*. [29] elucidated the overall structure of human MCU under low Ca^2+^ conditions, providing direct visualization. An interesting phenomenon with the human MCU complex is that under resting conditions or low [Ca^2+^]c, the MICU1–MICU2 complex blocks the MCU pore, thereby preventing Ca^2+^ influx into the mitochondria. In contrast, when cells are stimulated or the [Ca^2+^]c rises above a certain threshold, the complex allows the MCU pore to open, enabling mCa^2+^ uptake [29, 37]. Wu *et al*. [30] summarized three representative models proposed to describe the interaction between these two regulators.

Gherardi *et al*. [40] reported that oleuropein can bind to MICU1 to stimulate mCa^2+^ uptake and transiently increase mCa^2+^ levels within the physiological range, thereby promoting energy metabolism in both young and aged mice. Although the primary experiment focused on skeletal muscle, the same core components are also present in cardiomyocytes. Therefore, we speculate that a similar mechanism may apply to cardiomyocytes, although further experimental validation is required.

Another recent study from Zaglia *et al*. [41] found that overexpression of MCU enhances mCa^2+^ uptake, which exerts a positive effect on the heart’s adaptation to chronic pressure overload, and revealed the mechanism of this compensatory response: retrograde mCa^2+^/reactive oxygen species (ROS)/protein kinase B (Akt) signaling. This provides a potential therapeutic target for interventions aimed at preventing the progression from pathological cardiac hypertrophy to HF.

#### 2.1.3 Mitochondria-Associated Endoplasmic Reticulum Membranes

The endoplasmic/sarcoplasmic reticulum (ER/SR) and mitochondria serve as key Ca^2+^ reservoirs. Researchers have discovered a connecting structure between these organelles, referred to as “mitochondria-associated endoplasmic reticulum membranes” (MAMs) [21, 42]. This critical functional platform provides an efficient path for Ca^2+^ transport between the two organelles [43]. Ca^2+^ originating in the ER is precisely conveyed to the mitochondria through MAMs, then transduced into physiological signals that regulate several fundamental cellular processes, including energy metabolism and apoptosis [44, 45].

**Fig. 1.**
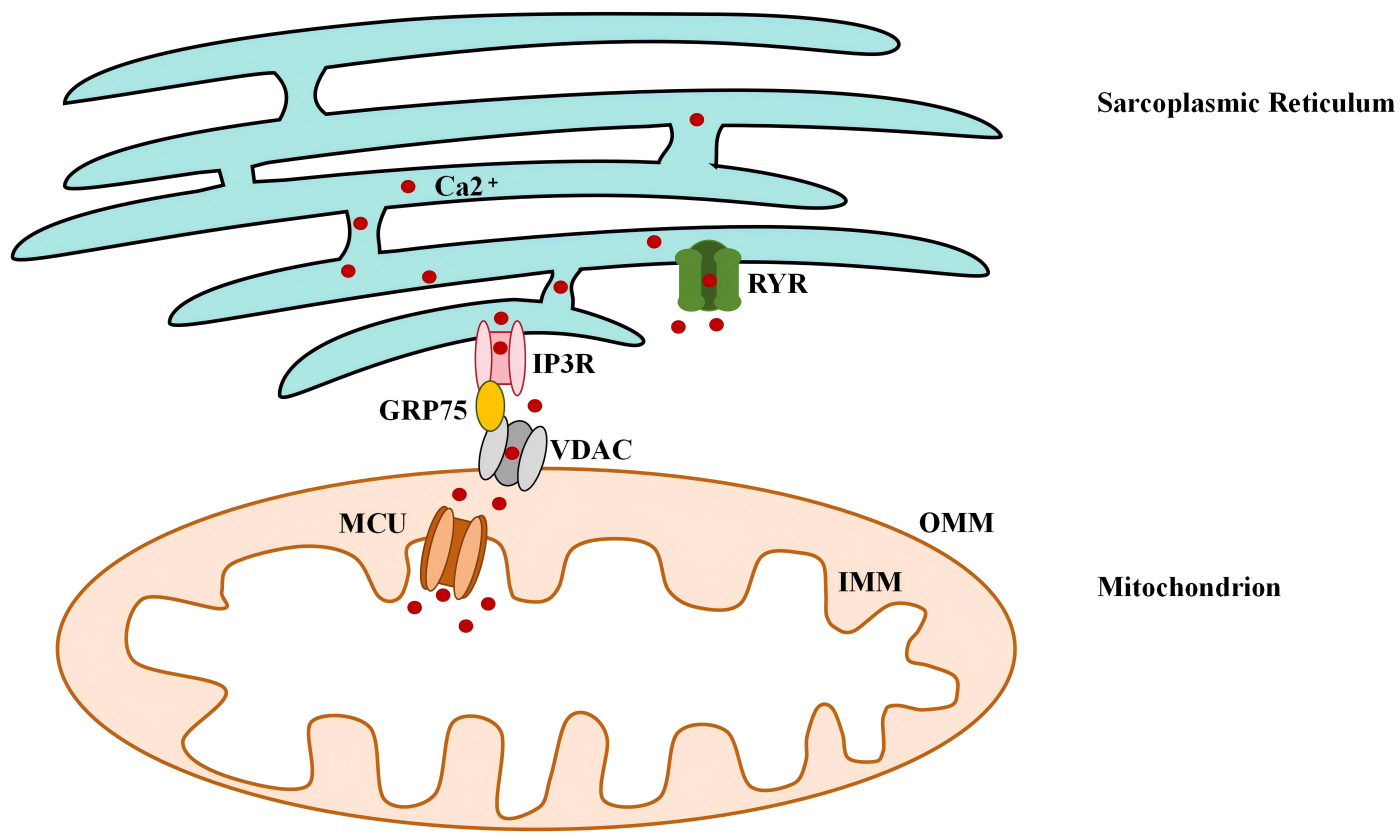
**The Ca^2+^ flux in MAMs of cardiomyocyte**. RyR, ryanodine receptor; IP3R, inositol 1,4,5-trisphosphate receptor; GRP75, glucose-regulated protein 75; MCU, mitochondrial Ca^2+^ uniporter; VDAC, voltage-dependent anion channel; OMM, outer mitochondrial membrane; IMM, inner mitochondrial membrane; MAMs, mitochondria-associated endoplasmic reticulum membranes. First, Ca^2+^ is released from the SR through IP3R or RyR. Next, the released Ca^2+^ crosses the OMM via VDAC into the IMS. Finally, Ca^2+^ is transported into the mitochondrial matrix through the MCU.

The mitochondrial uptake through MAMs of Ca^2+^ released from the SR involves three main steps [46] (see Fig. 1).

These proteins do not function independently. Some protein complexes play an important regulatory role in the cardiovascular system, with IP3R1 serving as a key regulator in the development of cardiac hypertrophy [47, 48].

MAMs play a pivotal role in the regulation of cardiovascular function. When the integrity of MAMs is disrupted, the ability of mitochondria to buffer cCa^2+^decreases, leading to abnormal elevation of the [Ca^2+^]c and ultimately promoting the progression of pathological cardiac hypertrophy and HF [49]. The structure and function of MAMs are frequently impaired in the context of HF. This impairment compromises mitochondrial energy metabolism, further exacerbates cardiomyocyte death, and accelerates disease progression [44, 50]. Mutations in the *RYR2* gene in mice have been shown to increase the Ca^2+^ flux into mitochondria via MAMs, thereby triggering HF [51]. Additionally, certain proteins located on MAMs exert a protective effect on cardiomyocytes, which may play a positive role in delaying the onset and progression of HF [52]. MAMs are also closely associated with the production of ROS, as well as with the occurrence of oxidative stress.

Gong *et al*. [53] reported that Mtus1A improves mitochondrial function in cardiomyocytes by preserving ER-mitochondria communication, suggesting it has potential as a therapeutic target following myocardial infarction.

### 2.2 Mitochondrial Calcium Efflux System

The efficient export of Ca^2+^ is essential in order to maintain mCa^2+^ homeostasis. This efflux is mediated by specialized transporters, notably the Na⁺/Ca^2+^/Li⁺ exchanger (NCLX), which serves as the primary mechanism for Ca^2+^ extrusion in cardiac mitochondria [28, 54, 55].

#### 2.2.1 Na⁺/Ca^2^⁺/Li⁺ Exchanger

NCLX plays a central role in maintaining cCa^2+^ homeostasis. It not only exports Ca^2+^ from the mitochondria, but also transfers Ca^2+^ to the SR. Studies have shown that NCLX is spatially and functionally coupled with the SR/ER Ca^2+^ ATPase. Mathematical models based on the structural characteristics of these proteins provide valuable insights into how the inhibition of NCLX impacts the re-uptake of SR Ca^2+^ in HL-1 cardiomyocytes [56]. The balance of mCa^2+^ uptake depends on the coordinated action of NCLX and other mitochondria-localized proteins, which facilitate the timely transport of Ca^2+^ into the cytosol [57]. Research has also shown that inhibition of NCLX decreases the generation of ROS induced by hypoxia, without altering mitochondrial respiratory function. This highlights the specific role of NCLX in the mechanism of ROS generation [58]. A recent study from Fan *et al*. [59] has revealed that NCLX serves a transport function as an H^+^/Ca^2+^ exchanger.

#### 2.2.2 Mitochondrial Permeability Transition Pore

An optimal m[Ca^2+^] is essential for cardiomyocyte function, as Ca^2+^ activates metabolic enzymes to meet cellular energy demands. However, excessive m[Ca^2+^] can trigger opening of the mPTP in the IMM, mediating the release of Ca^2+^. While transient opening of the mPTP regulates m[Ca^2+^] and energy metabolism, prolonged opening leads to the collapse of membrane potential, inhibition of ATP synthesis, and ultimately to cell death [23, 55, 60]. NCLX plays an important role in combating Ca^2+^ overload [55]. In adult mouse models, specific knockdown of NCLX in the heart results in mCa^2+^ overload, leading to severe cardiac dysfunction. In contrast, the overexpression of NCLX can effectively rescue cell death and prevent HF in post-myocardial infarction models [23]. Recent studies suggest that NCLX may be a potential therapeutic target, particularly for the prevention of cardiac hypertrophy, cardiogenic sudden death, and other cardiovascular diseases [61, 62]. Furthermore, recent work confirms that NCLX is a key physiological pathway for mCa^2+^ efflux in cardiomyocytes [63, 64].

The mPTP is also considered to be associated with HF, although the mechanism underlying its activation remains incompletely understood [65]. Albanese *et al*. [66] reported finding new molecules that can inhibit opening of the mPTP in an *in vitro* cardiac model.

Furthermore, studies have found that naringenin and melatonin may inhibit the opening of mPTP. This provides an opportunity to study their potential as therapeutic agents for diseases associated with mitochondrial dysfunction linked to mPTP opening [67, 68].

## 3. Pathological Associations Between Mitochondrial Calcium Dysregulation and Heart Failure

**Fig. 2.**
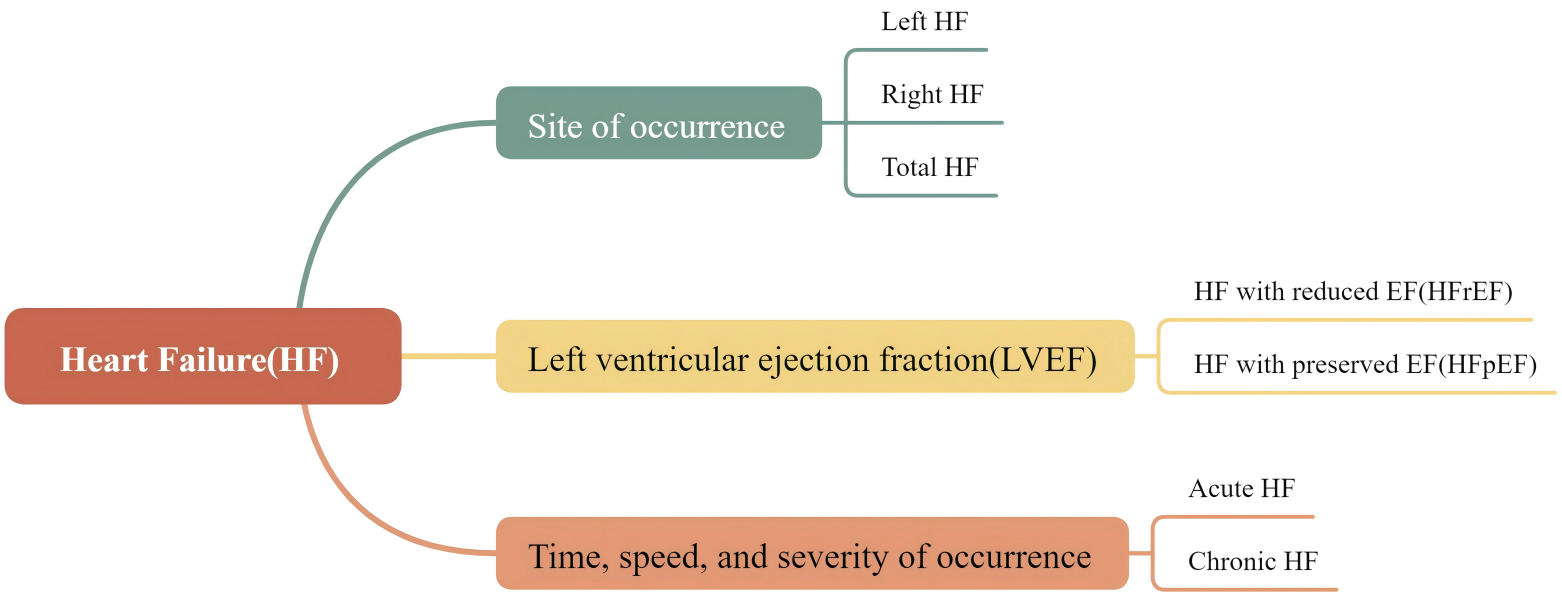
**The typical categories of HF**. HF is classified into different types based on various indicators. Based on the site of occurrence, it can be classified into left HF, right HF, and total HF; based on LVEF, it can be categorized as HFrEF and HFpEF; based on the time, speed, and severity of occurrence, it can be divided into acute HF and chronic HF.

HF is caused by impaired cardiac pumping function, resulting in the inability to meet the body’s fundamental metabolic demands. It is classified into different types based on various indicators, as illustrated in Fig. 2.

HFrEF and HFpEF appear to be different in terms of myocardial mCa^2+^ cycling. m[Ca^2+^] is decreased in HFrEF, but elevated in HFpEF [69]. Although this may seem counterintuitive, in both cases the underlying mechanisms ultimately lead to HF. Therapies targeting mCa^2+^ may have different effects between HFrEF and HFpEF. For example, some researchers suggest that: overexpression of MCU increases the m[Ca^2+^] and improves the HFrEF phenotype. While it has negligible effects on HFpEF. Currently, there appears to be insufficient clinical evidence for mCa^2+^-targeted therapies in these two types of HF.

### 3.1 Energy Metabolism Dysfunction

The basis of cardiac metabolism lies in the production and utilization of ATP, a high-energy molecule that is essential for cardiac contraction, basal metabolism, and the maintenance of normal cardiac function [70]. The heart is a high energy-demanding organ and consumes significant amounts of energy with each contraction. A continuous supply of ATP is therefore critical, and any disruption in metabolic pathways can have profound consequences for cardiac function [70, 71, 72]. Approximately 95% of the ATP utilized by the heart is derived from mitochondrial oxidative metabolism. Mitochondria are therefore essential for maintaining the internal energy supply and ensuring optimal cellular function [10, 73, 74]. Heart dysfunction can arise from a variety of factors, but most are intricately linked to mitochondrial damage. Mitochondrial dysfunction serves as a central mechanism in the development of HF by compromising the energy supply [71, 75].

**Fig. 3.**
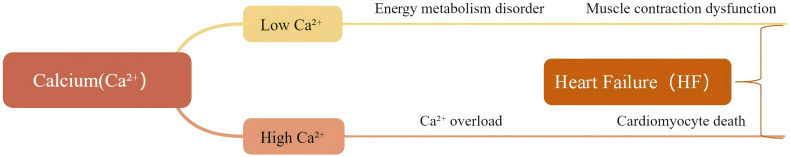
**The relationship between Ca^2+^ and HF**. Different Ca^2+^ levels may lead to HF. Low Ca^2+^ can cause impaired energy production, thereby affecting muscle contraction and ultimately resulting in HF. However, high Ca^2+^ can induce Ca^2+^ overload, leading to cardiomyocyte death and eventually triggering HF.

mCa^2+^ is a key signaling molecule in the regulation of ATP synthesis [72, 75]. Additionally, mCa^2+^ can stimulate the activity of key enzymes, thereby facilitating efficient ATP production [76, 77]. In response to increased myocardial workload, mitochondria accumulate Ca^2+^ and promptly upregulate energy metabolism to ensure an adequate energy supply for excitation-contraction coupling [78]. Maintaining mCa^2+^ homeostasis is therefore crucial for regulating myocardial metabolism. The relationship between Ca^2+^ and HF mentioned above is summarized in Fig. 3 below.

### 3.2 Oxidative Stress and Cell Death

A central pathological feature of HF is the widespread dysregulation of cCa^2+^ homeostasis. The perturbation of Ca^2+^ homeostasis precipitates mCa^2+^ overload, further exacerbating mitochondrial dysfunction and oxidative stress, and accelerating the progression of HF [79].

Mitochondria are the primary site of ROS production in cardiomyocytes. Under physiological conditions, ROS are produced at low levels and function as signaling molecules in cellular regulation. They can be effectively neutralized by endogenous antioxidant systems. However, when ROS production exceeds the clearance capacity, oxidative stress ensues, leading to significant damage to myocardial structure and function [80]. In HF models, elevated levels of ROS are detected within the mitochondrial matrix, accompanied by the depletion of antioxidant reserves [71, 78].

**Fig. 4.**
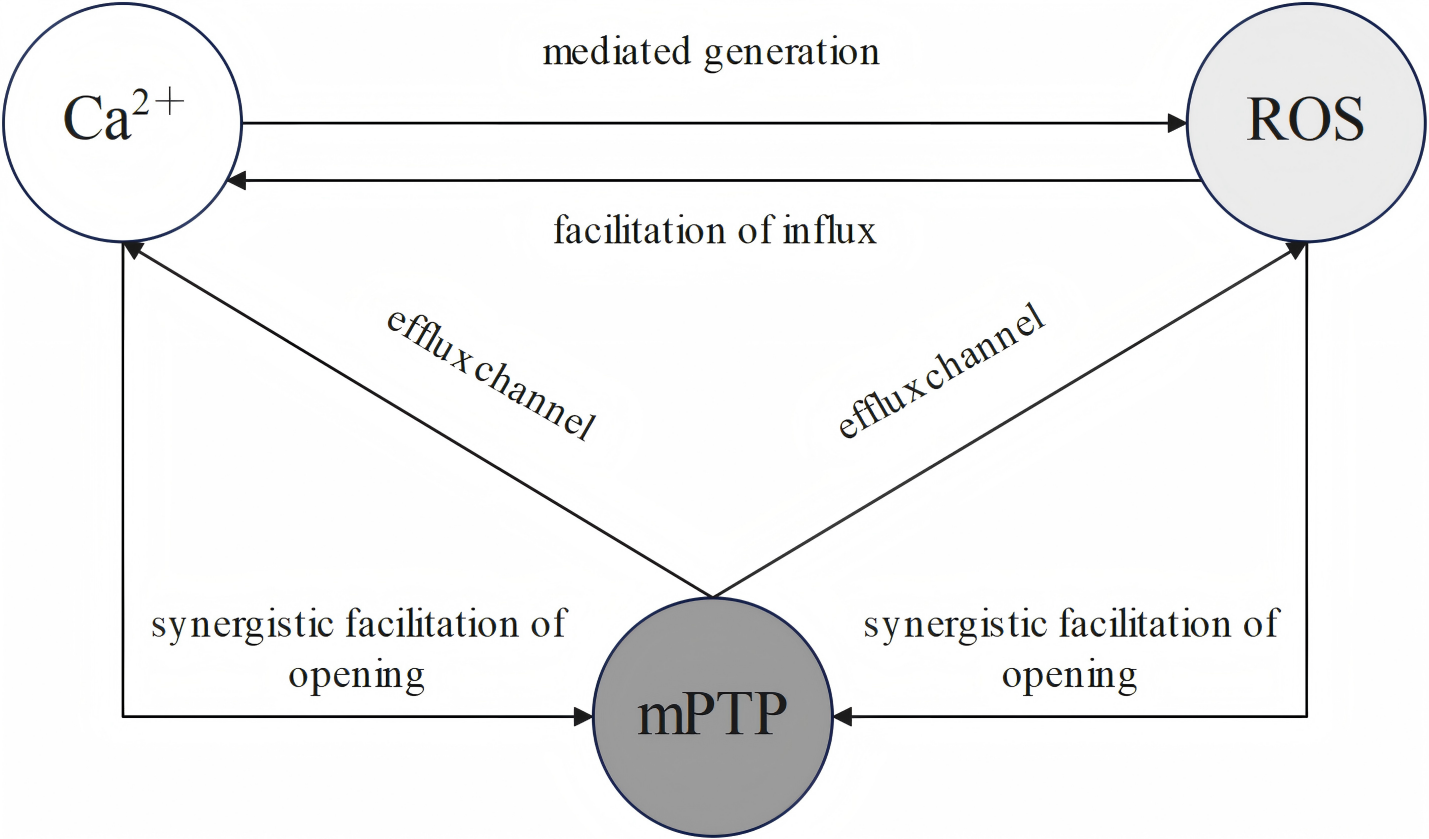
**Schematic representation of the interplay among ROS, Ca^2+^, with mPTP**. ROS, reactive oxygen species; mPTP, mitochondrial permeability transition pore. The generation of ROS is critically dependent on Ca^2+^, and ROS can facilitate Ca^2+^ influx into the mitochondria. Ca^2+^ and ROS promote the opening of mPTP, and its opening leads both Ca^2+^ and ROS to efflux.

Impaired mCa^2+^ uptake represents a critical event in the pathogenesis and progression of HF. This defect weakens the reducing capacity of critical coenzyme pairs, resulting in disruption of redox homeostasis. The imbalance in redox status leads to insufficient ATP production and triggers oxidative stress with a substantial accumulation of ROS. Excess ROS further activates calmodulin-dependent protein kinase II, thereby exacerbating the dysregulation of Ca^2+^ to form a vicious cycle [69, 75]. A close, bidirectional regulatory relationship exists among Ca^2+^, ROS with mPTP (see Fig. 4).

Opening of the mPTP increases permeability of the IMM, which is normally tightly regulated by several factors. mCa^2+^ overload and ROS accumulation are the primary triggers for mPTP opening [81]. A halt in ATP synthesis occurs under conditions of sustained opening [70, 75, 82]. Furthermore, mitochondria undergo swelling and rupture of the OMM, resulting in the release of pro-apoptotic factors that can drive either apoptosis or necrosis.

## 4. Mitochondria-Targeted Therapy

Antioxidants, mitochondria-targeted agents, and cardioprotective drugs have all shown potential in improving mitochondrial function [83]. The regulation of mCa^2+^ channel proteins might be a promising strategy for mitochondria-targeted therapy.

The MCU serves as the primary pathway for Ca^2+^ entry into the mitochondria. However, studies have shown that excessive reliance on MCU-mediated mCa^2+^ uptake is detrimental for adaptation by the heart under sustained hemodynamic stress, and may also lead to cardiomyocyte injury [84]. Consequently, the MCU is recognized as a potential therapeutic target [85, 86].

### 4.1 MCU Inhibitors

Ruthenium-based compounds are the most extensively studied class of MCU inhibitors. Ru360 has been shown to effectively block mCa^2+^ uptake and confer beneficial effects in animal models. However, its clinical translation remains limited due to challenges such as poor delivery efficiency, high toxicity, and off-target effects [87, 88]. To overcome these limitations, novel ruthenium-based compounds have been developed, such as Ru265. Compared to Ru360, Ru265 exhibits enhanced cell membrane permeability and reductive stability, while also effectively inhibiting mCa^2+^ influx in intact cells [89, 90]. Fluorescent probe–type MCU inhibitors, such as RuOCou, have both therapeutic and imaging functions, thus offering new possibilities for theranostic strategies [91].

Berberine, a Food and Drug Administration (FDA)-approved drug with a well-established safety profile, was recently reported to be an effective inhibitor of MCU. Mechanistically, berberine blocks excessive mCa^2+^ uptake via disrupting the interaction between MCU and EMRE, providing a potential strategy for the treatment of diseases associated with an imbalance of mCa^2+^ homeostasis [92].

DS16570511 (DS) can effectively suppress the activity of the MCU complex and attenuate mCa^2+^ influx [93], but its precise mechanism remains incompletely understood. Current experimental evidence indicates that the effects of DS vary across different cells and mitochondria [94].

### 4.2 Elamipretide

Elamipretide is a highly selective, mitochondria-targeted tetrapeptide. It has attracted considerable attention as a therapeutic candidate due to its ability to specifically bind to cardiolipin within the IMM. Elamipretide prevents aberrant opening of the mPTP and blocks the initiation of apoptotic signaling [95, 96, 97]. In preclinical models and early clinical trials, Elamipretide has been shown to attenuate some diseases with mitochondrial dysfunction, such as aging-associated sarcopenia and HF [96, 97]. It also shows therapeutic potential in the treatment of rare disorders, including Barth syndrome [98]. Preliminary clinical data suggests that Elamipretide exhibits a favorable safety profile, and may improve cardiac hemodynamic parameters at higher doses. However, the long-term efficacy and safety of this agent remain to be further validated [99]. The research progress of some drugs is shown in Table 2 (Ref. [87, 91, 92, 93, 94, 98, 100]).

**Table 2.** **Research progress of several drugs**.

Category	Drug	Clinically Validated	Preclinical models
MCU inhibitor	Ru360	No	TBI rats [87]
	Ru265	No	HEK293T cells [100]
	RuOCou	No	HeLa cells [91]
	Berberine	Yes [92]	-
	DS	No	Rat liver cells [94] and cortical neurons [93]
Tetrapeptide	Elamipretide	Yes [98]	-

MCU, mitochondrial Ca^2+^ uniporter; DS, DS16570511; TBI, traumatic brain injury.

Luongo *et al*. [101] and Garbincius *et al*. [54] demonstrated that overexpression of NCLX has a beneficial effect in delaying the progression of HF. Transgenic mice with a neuron-specific knockout of the *NCLX* gene recapitulated an Alzheimer’s disease-like pathology [102]. While these results support the targeting of NCLX activity as a promising therapeutic strategy for various diseases, its clinical translation requires a better understanding of the mechanisms regulating NCLX function. The latest findings from Fan *et al*. [59] on NCLX may provide clues for its clinical translation.

In addition to the drugs related to mCa^2+^ previously discussed, several agents that target mitochondria in HF models are shown in Table 3 (Ref. [103, 104, 105, 106, 107, 108]). Although these strategies relate to mitochondria, they have no clear association with mCa^2+^.

**Table 3.** **Mitochondria-targeted drugs in HF models**.

Authors	Strategy	Agent	Outcome	HF model
Jia *et al*. [103]	Endogenous anti-oxidation	Aerobic exercise	Attenuation of mitochondrial injury	Male mice, HF induced by a left ventricular pressure overload established by TAC
Bradley *et al*. [104]	Mitochondrial DNA repair	Exscien1-III	Substantial protection against myocardial ischemia	Male C57/BL6J mice, HF induced by myocardial ischemia-reperfusion injury and TAC
Zhang *et al*. [105]	Inhibition of ferroptosis	Resveratrol	Decelerated fibrosis progression	Mice, HF with aortic coarctation in Sirt1 knockout
Park *et al*. [106]	Mitochondrial di-carbonyl scavenging	MitoGamide	Improved diastolic function	Akita mice
Kim *et al*. [107]	Mitochondrial-targeted anti-oxidant	MitoQ	Improvement of cardiac mitochondrial network integrity	C57BL/6J mice, HF induced by AAC
Filipiak *et al*. [108]	Dietary therapy	Coenzyme Q10	Effective production of ATP	Patients

HF, heart failure; TAC, transverse aortic constriction; AAC, ascending aortic constriction; ATP, adenosine triphosphate.

## 5. Conclusion

Mitochondrial dysfunction is closely associated with the pathogenesis of various common diseases, and dysregulation of mCa^2+^ is a critical factor in the pathological progression of HF. In order to advance novel therapeutic approaches aimed at mitigating the burden of HF, it is imperative to gain a deeper understanding of the molecular mechanisms governing mCa^2+^ uptake, along with its associated regulatory pathways [79, 83]. Importantly, most of the aforementioned drug candidates have yet to enter clinical trials, and their safety and efficacy profiles remain to be determined. The dosage of medication should be taken into consideration accounting for health and safety. Furthermore, the potential cytotoxicity of some compounds must be considered, as they could exacerbate HF by damaging cardiomyocytes. Besides, delivery and off-target effects remain challenges that need to be addressed. Research has found that acute deletion of NCLX in mature cardiomyocytes leads to mCa^2+^ overload and HF [109], whereas cardiac-specific overexpression of NCLX enhances mCa^2+^ clearance and prevents HF [101]. Thus, *NCLX* gene therapy may represent a promising strategy for treating HF.
